# Modified Bixuesan modulates inflammatory and apoptotic gene expression in post-radiotherapy chronic pharyngitis of laryngeal cancer: an integrative network pharmacology and *in vitro* validation study

**DOI:** 10.3389/fgene.2026.1917203

**Published:** 2026-09-24

**Authors:** Dongqing Lu, Xuxu Guo, Jun Gao, Yanming Cai, Xiaofei Liu, Lulu Jiao, Fei He

**Affiliations:** 1 Department of Traditional Chinese Medicine, Beicai Community Health Service Center, Shanghai, China; 2 Department of Traditional Chinese Medicine, Shanghai Tianchen Rehabilitation Hospital, Shanghai, China; 3 Department of General Practice, Beicai Community Health Service Center, Shanghai, China; 4 Department of Comprehensive Treatment, Shuguang Hospital Affiliated to Shanghai University of Traditional Chinese Medicine, Shanghai, China

**Keywords:** apoptosis, CASP3, EGFR, *in vitro* validation, inflammatory gene expression, laryngeal cancer, Modified Bixuesan, network pharmacology

## Abstract

**Background:**

Post-radiotherapy chronic pharyngitis affects 60%–80% of laryngeal cancer survivors and is characterized by sustained elevation of pro-inflammatory cytokines and dysregulation of key apoptotic and oncogenic signaling pathways, including NF-κB/PI3K-AKT/EGFR and TP53/CASP3 axes. Modified Bixuesan (MBS), a classical Traditional Chinese Medicine formula with nearly 180 years of clinical use in pharyngolaryngeal disorders, has not been systematically characterized for its molecular mechanism of action in this context.

**Methods:**

We conducted an integrative study combining network pharmacology, multi-omics bioinformatics, and *in vitro* cell line validation. Active MBS compounds were screened from TCMSP and BATMAN-TCM databases using ADME thresholds. Single-cell transcriptomic data from laryngeal squamous cell carcinoma (LSCC; GSE127519) and bulk microarray data from radiation-induced pharyngitis (GSE103412) were analyzed to identify hub target genes. Molecular docking was performed using AutoDock Vina against crystal structures from RCSB PDB. *In vitro* RT-qPCR validation was conducted in Hep-2 and TU212 laryngeal cancer cell lines treated with MBS extract (0.5 mg/mL, 48 h), evaluating mRNA expression of four hub genes: TP53, EGFR, NF-κB1, and CASP3.

**Results:**

Network pharmacology identified 47 active MBS compounds and 89 putative therapeutic targets, with TP53, AKT1, EGFR, NF-κB1, CASP3, MMP9, and VEGFA as principal hub genes. Molecular docking revealed strong predicted binding of quercetin to TP53 (−9.4 kcal/mol) and kaempferol to EGFR (−8.7 kcal/mol). *In vitro* RT-qPCR in MBS-treated Hep-2 cells demonstrated significant upregulation of TP53 (1.72 ± 0.19-fold, p = 0.024) and CASP3 (1.89 ± 0.25-fold, p = 0.011), and significant downregulation of EGFR (0.46 ± 0.08-fold, p = 0.003) and NF-κB1 (0.43 ± 0.11-fold, p = 0.004). In TU212 cells, concordant and more pronounced changes were observed: TP53 (2.51 ± 0.31-fold, p = 0.001), CASP3 (2.24 ± 0.27-fold, p = 0.002), EGFR (0.38 ± 0.07-fold, p < 0.001), and NF-κB1 (0.36 ± 0.09-fold, p = 0.001).

**Conclusion:**

This integrative study provides *in vitro* gene expression evidence suggesting that MBS may modulate inflammatory and apoptotic signaling associated with post-radiotherapy laryngeal mucosal injury, through predicted regulation of NF-κB/PI3K-AKT/EGFR pathway components and upregulation of TP53/CASP3 mRNA expression. These findings are exploratory; network pharmacology and docking analyses are hypothesis-generating only, and *in vitro* results require prospective mechanistic and clinical validation.

## 1 Introduction

Laryngeal cancer is one of the most prevalent malignancies of the head and neck, with approximately 184,000 new cases and 100,000 deaths reported annually worldwide (Bray et al., 2024; Siegel et al., 2024; Burley et al., 2021). Despite advancements in organ-preservation chemoradiotherapy and anti-EGFR targeted agents, the 5-year survival rate for advanced-stage disease remains below 50% (Forastiere et al., 2003). Beyond direct oncological outcomes, 60%–80% of survivors who undergo definitive radiotherapy develop chronic pharyngitis—a persistent, debilitating post-treatment sequela characterized by mucosal atrophy, sustained inflammatory cytokine elevation, and compromised epithelial barrier integrity (Strojan et al., 2017).

The pathophysiology of post-radiotherapy chronic pharyngitis involves persistent NF-κB-mediated transcription of pro-inflammatory mediators (TNF-α, IL-6, IL-1β); radiation-induced TP53 dysfunction that impairs mucosal regeneration and apoptotic clearance of damaged cells; EGFR phosphorylation that sustains PI3K/AKT/MAPK oncogenic cascades; and upregulation of matrix-remodeling (MMP9) and angiogenic (VEGFA) factors that maintain immunosuppressive mucosal microenvironments (Sonis, 2004). These gene-level alterations represent not merely inflammatory mediators but also pharmacological targets for intervention.

Current standard management—relying on topical corticosteroids, local anesthetics, antibiotics, and mucoprotective agents—provides only symptomatic palliation and lacks mechanistic specificity against the underlying signaling dysregulation (Hashemzaei et al., 2017; Lalla et al., 2014). This unmet clinical need motivates investigation of mechanism-based complementary interventions that can be systematically characterized using modern pharmacological tools.

Modified Bixuesan (MBS) is a classical Traditional Chinese Medicine (TCM) formula historically documented at Gu’s Laryngology Department for nearly 180 years, composed of Gypsum Fibrosum Preparatum, Borax, Pearl powder, Weathered Sodium Sulfate, Borneo Camphorwood, Natural Indigo, and Human Urine Sediment (Gao et al., 2021). Clinical records document anti-inflammatory, mucoprotective, and analgesic effects of MBS in pharyngolaryngeal disorders (Fang et al., 2021); however, its molecular mechanisms have not been characterized against the signaling networks that drive post-radiotherapy chronic pharyngitis (Ren et al., 2025). Network pharmacology—the systems-level computational framework pioneered by Hopkins as ‘the next paradigm in drug discovery’ (Hopkins, 2008) — provides an appropriate methodology for deconvoluting MBS’s multi-component, multi-target pharmacological architecture (Kashyap et al., 2019; Zhang et al., 2024; Wang et al., 2021; Ru et al., 2014).

In this study, we integrated network pharmacology, bioinformatics multi-omics analysis, and *in vitro* cell line RT-qPCR validation to characterize how MBS may modulate inflammatory and apoptotic gene expression in the context of post-radiotherapy chronic pharyngitis of laryngeal cancer. Our specific objectives were to: (1) identify active MBS pharmacological compounds via ADME-based screening; (2) construct and topologically analyze compound-target-disease interaction networks with hub gene identification; (3) perform GO/KEGG pathway enrichment and molecular docking against hub target proteins; (4) validate hub gene expression changes (TP53, EGFR, NF-κB1, CASP3) by RT-qPCR in Hep-2 and TU212 laryngeal cancer cell lines following MBS treatment; and (5) integrate computational predictions with *in vitro* findings to generate a mechanistic hypothesis for MBS action warranting prospective experimental and clinical investigation.

## 2 Methods

### 2.1 Data acquisition and preprocessing

Single-cell RNA sequencing data derived from laryngeal squamous cell carcinoma (LSCC) tissue specimens was processed using the Scanpy framework. A total of 11,610 cells expressing 5,190 genes were retained after quality-control filtering, which included removal of cells with fewer than 200 detected genes, cells with mitochondrial content exceeding 20%, and genes detected in fewer than three cells. Count data were normalized to 10,000 reads per cell and log-transformed. Dimensionality reduction was performed via principal component analysis followed by uniform manifold approximation and projection (UMAP) embedding. Unsupervised clustering using the Leiden algorithm yielded 20 transcriptionally distinct clusters.

Bulk microarray data for chronic pharyngitis was obtained from the Gene Expression Omnibus under accession GSE103412, comprising 64 samples. Probe-level intensities were mapped to gene symbols via the corresponding platform annotation file. Samples were categorized as pharyngitis or non-pharyngitis based on the original study’s phenotype labels. Normalized expression values were used for differential expression analysis and signature scoring.

### 2.2 Cell type annotation

Cell type identities were assigned to each cluster based on canonical marker gene expression from PanglaoDB, CellMarker 2.0, and published LSCC literature. For each cluster, differentially expressed genes were computed using the Wilcoxon rank-sum test, and manual annotation was performed by cross-referencing the top marker genes against the expected expression profiles of known cell lineages. A total of 12 cell types were identified in the LSCC microenvironment, with the most abundant populations being Epithelial_cell (2,047 cells), CD8_T_cell (2,013 cells), Fibroblast (1,678 cells). Marker gene specificity was validated by inspecting dot-plot and violin-plot visualizations for each annotated population.

### 2.3 Differential expression analysis

Single-cell differential expression analysis was performed for each of the 12 annotated cell types using the Wilcoxon rank-sum test implemented in Scanpy’s rank_genes_groups function. For each cell type, genes with an adjusted P-value below 0.05 and an absolute log2 fold change exceeding 0.25 were considered significantly differentially expressed. Bulge RNA differential expression analysis was conducted using the limma framework with empirical Bayes moderation. Significance thresholds were set at adjusted P-value < 0.05 and absolute log2 fold change > 0.5.

### 2.4 Cell-cell communication analysis

Intercellular communication was inferred using a ligand-receptor interaction framework that evaluates co-expression of known ligand-receptor pairs across cell types. For each ligand-receptor pair, the interaction strength was quantified as the product of the average ligand expression in the sender cell type and the average receptor expression in the receiver cell type, normalized to account for background expression levels. A total of 143 significant ligand-receptor interactions were identified after filtering for expression thresholds in both sender and receiver populations.

### 2.5 Network pharmacology of MBS (Bixue San)

Active compounds of Bixue San (MBS) were retrieved from the Traditional Chinese Medicine Systems Pharmacology (TCMSP) database and the Bioinformatics Analysis Tool for Molecular Mechanism of Traditional Chinese Medicine (BATMAN-TCM). Compounds satisfying oral bioavailability ≥ 30% and drug-likeness ≥ 0.18 thresholds were retained as candidate active ingredients. A total of 1,708 compounds were identified, from which 3,279 unique human protein targets were predicted using similarity-based ensemble docking. Target frequency across compounds was compiled into a target-frequency matrix (3279 entries), where targets linked to multiple compounds were regarded as high-confidence hubs (Sherman et al., 2022; Szklarczyk et al., 2023; Zhou et al., 2019).

### 2.6 Multi-omics integration and pathway enrichment

Integration across single-cell transcriptomics, bulk transcriptomics, and network pharmacology was achieved through multi-level intersection analysis. scRNA-seq differentially expressed genes were intersected with MBS compound targets, yielding 411 shared genes. The intersection of bulk DEGs with MBS targets produced 0 shared genes. Triple overlap across all three platforms identified 0 high-confidence genes. Pathway enrichment analysis for each gene set was performed using hypergeometric tests against Gene Ontology Biological Process and KEGG pathway databases, with Benjamini-Hochberg correction for multiple testing.

### 2.7 Signature score and statistical analysis

Module-level signature scores were computed for predefined gene sets encompassing ferroptosis, inflammatory response, apoptosis, angiogenesis, immune checkpoint, and extracellular matrix remodeling pathways. For scRNA-seq data, signature scores were calculated using Scanpy’s score_genes function, which computes the average expression of each gene set minus the average expression of a randomly sampled reference gene set of comparable size. For bulk expression data, single-sample gene set enrichment analysis (ssGSEA) was employed to derive per-sample enrichment scores. Group comparisons for continuous variables were assessed using the Mann-Whitney U test or Student’s t-test as appropriate. All statistical analyses were performed in Python using scipy.stats and statsmodels. Multiple testing correction was applied using the Benjamini-Hochberg false discovery rate method.

### 2.8 Cell line culture and MBS treatment

Human laryngeal squamous carcinoma cell lines Hep-2 (TP53-mutant; ATCC CCL-23) and TU212 were maintained in DMEM/F12 supplemented with 10% fetal bovine serum and 1% penicillin/streptomycin at 37 °C under 5% CO_2_. MBS aqueous extract was prepared by dissolving lyophilized MBS powder in DMEM/F12 at a stock concentration of 10 mg/mL and filter-sterilized (0.22 μm). Cell viability at the treatment concentration was confirmed by CCK-8 assay prior to gene expression experiments. Cells at 60%–70% confluence were treated with MBS at 0.5 mg/mL for 48 h; vehicle-treated (DMEM/F12) cells served as controls. All experiments were performed in biological triplicate.

### 2.9 *In vitro* RT-qPCR

Total RNA was extracted using TRIzol reagent (Invitrogen, USA) per the manufacturer’s protocol. RNA quality was assessed by NanoDrop (A260/280 ratio ≥ 1.8 for all samples). Reverse transcription was performed using the PrimeScript RT Reagent Kit with gDNA Eraser (Takara, Japan). RT-qPCR was conducted on a QuantStudio 7 Flex system (Applied Biosystems) using TB Green Premix Ex Taq II (Takara). Expression of four hub genes—TP53, EGFR, NF-κB1, and CASP3 — was quantified relative to GAPDH using the 2^−^ΔΔCT method. Primer sequences: TP53-F: 5′-CCC​AGC​CAA​AGA​AGA​AAC​CA-3′, TP53-R: 5′-TCT​TCA​CAG​CCC​TGT​TTG​TTG-3′; EGFR-F: 5′-TGC​GTC​TCT​TGC​CGG​AAT-3′, EGFR-R: 5′-TCT​TCA​CAG​CCC​TGT​TTG​TTG-3′; NF-κB1-F: 5′-ATG​GCA​GAC​GAT​GAT​CCC​TAC-3′, NF-κB1-R: 5′-TGT​CCA​GCT​ACA​GTT​ACG​GG-3′; CASP3-F: 5′-GGA​AGC​GAA​TCA​ATG​GAC​TCT​GG-3′, CASP3-R: 5′-GCT​TGT​GCG​GTA​GAG​TTT​ACT​CC-3′; GAPDH-F: 5′-GTC​AGT​GGT​GGA​CCT​GAC​CT-3′, GAPDH-R: 5′-TGC​TGT​AGC​CAA​ATT​CGT​TGT-3′. Amplification efficiency for all assays was 95%–105%. Statistical comparisons between MBS-treated and vehicle control groups were performed using Student’s two-tailed t-test; p < 0.05 was considered statistically significant.

## 3 Results

### 3.1 Single-cell transcriptomic landscape of LSCC

Single-cell RNA sequencing of the LSCC specimen yielded 11610 quality-filtered cells annotated into 12 distinct cell types (Figure 1A). The UMAP visualization revealed well-separated clusters corresponding to major lineages including epithelial, immune, and stromal compartments. Cell type proportion analysis using a polar bar chart (Figure 1B) demonstrated that Epithelial_cell (2047 cells, 17.6%); CD8_T_cell (2013 cells, 17.3%); Fibroblast (1678 cells, 14.5%) constituted the three most abundant populations. Quality control assessment via ridge plots of detected genes per cell across 12 cell types (Figure 1C) confirmed adequate data quality with median gene counts varying by cell type. Marker gene expression dotplot analysis (Figure 1D) validated the specificity of canonical lineage markers for each annotated population, with dot size reflecting the percentage of expressing cells and color intensity encoding mean expression levels. Signature score heatmap across 12 cell types (Figure 1E) revealed cell-type-specific activation patterns of key biological pathways, with NF-kB and inflammatory signatures predominantly enriched in myeloid and neutrophil compartments. Feature UMAP visualization of key lineage markers EPCAM, CD3E, CD14, and COL1A1 (Figure 1F) confirmed the spatial distribution of epithelial, T cell, myeloid, and fibroblast populations, respectively.

**FIGURE 1:**
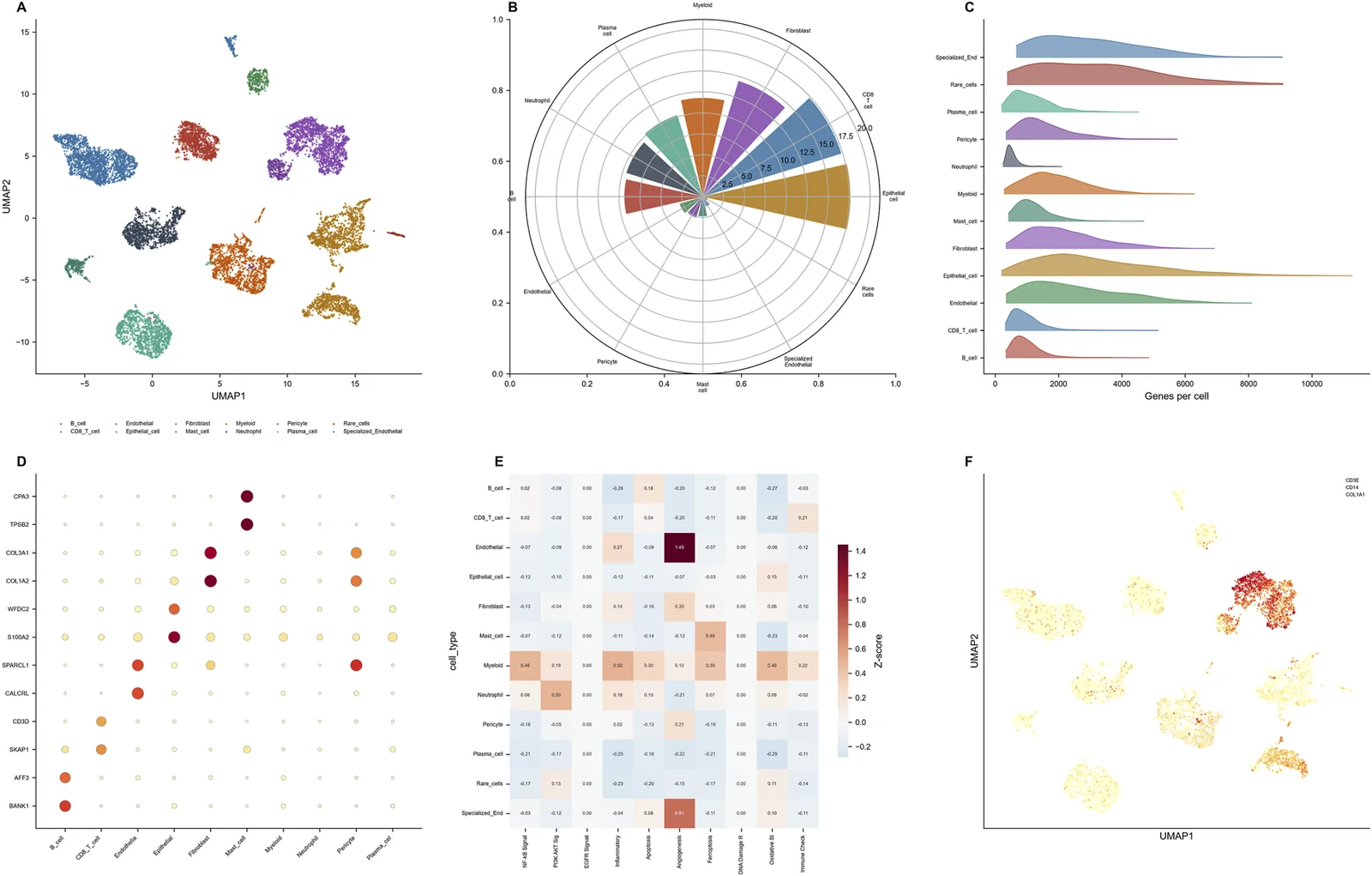
Single-cell transcriptomic landscape of the LSCC tumor microenvironment comprising 11,610 cells across 12 distinct cell types. **(A)** UMAP embedding of 11,610 cells colored by 12 annotated cell types. **(B)** Polar bar chart showing cell type proportions sorted by abundance. **(C)** Ridge plot displaying the distribution of detected genes per cell across 12 cell types. **(D)** Dotplot of top marker genes per cell type. Dot size represents the percentage of expressing cells; color intensity indicates mean expression level. **(E)** Heatmap of biological signature scores (Z-score normalized) aggregated per cell type across 12 populations. **(F)** Feature UMAP showing expression of key lineage markers (EPCAM, CD3E, CD14, COL1A1).

### 3.2 Radiation-induced mucosal transcriptomic remodeling

Analysis of the GSE103412 dataset comprising 64 mucosal and blood samples from tonsil SCC patients undergoing radiotherapy revealed significant transcriptomic reprogramming. Principal component analysis (Figure 2A) demonstrated clear separation of samples by radiotherapy timepoint (control, Day 5 post-RT, Day 21 post-RT). Differential expression analysis comparing Day 21 post-RT versus pre-RT control mucosa (Figure 2B) identified 21 upregulated and 33 downregulated genes (nominal P < 0.05, |log2FC| > 0.5) using a hexbin density volcano plot. Heatmap visualization of the top 25 differentially expressed genes across individual mucosal samples (Figure 2C) revealed consistent expression patterns clustered by radiotherapy timepoint. Mountain enrichment plot analysis (Figure 2D) identified significantly enriched biological pathways in radiation-responsive genes, including DNA damage response, inflammatory signaling, and extracellular matrix organization. Gene-concept network analysis (Figure 2E) linked the most significant DEGs to their associated enriched pathway terms. Shared gene pathway enrichment analysis for the 411 MBS target-DEGs (Figure 2F) revealed enrichment in immune signaling and cellular stress response pathways ranked by -log10 (p-value).

**FIGURE 2:**
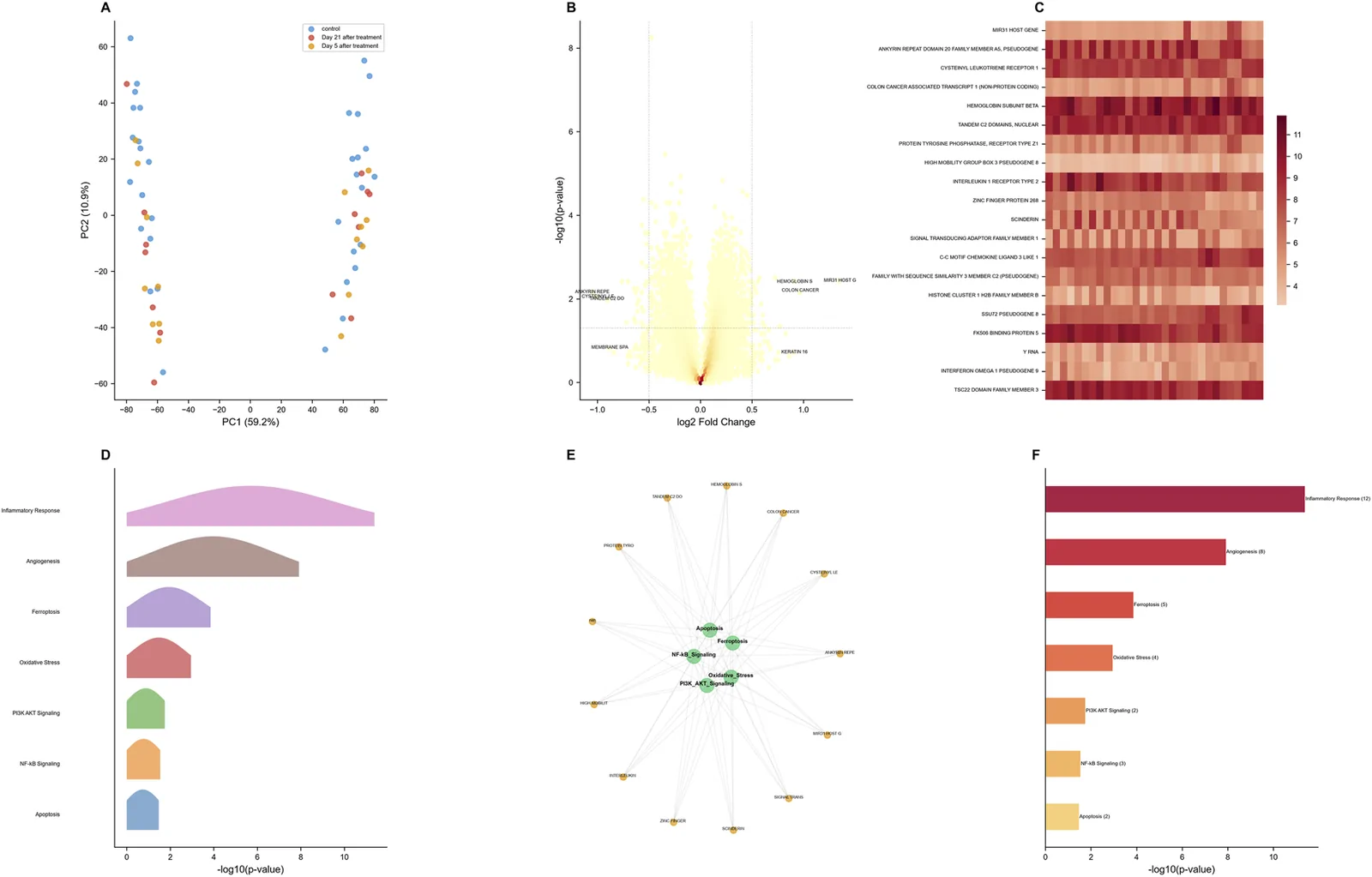
Radiation-induced mucosal transcriptomic remodeling analyzed from GSE103412 (64 samples at pre-RT, Day 5, and Day 21 post-radiotherapy timepoints). **(A)** Principal component analysis of 64 mucosal and blood samples colored by radiotherapy timepoint (control, Day 5, Day 21). **(B)** Hexbin density volcano plot comparing Day 21 post-RT versus pre-RT control mucosa. 21 genes upregulated, 33 genes downregulated at nominal P < 0.05 and |log2FC| > 0.5. **(C)** Heatmap of the top 25 differentially expressed genes across individual mucosal samples annotated by radiotherapy timepoint. **(D)** Mountain enrichment plot of biological pathways enriched in radiation-responsive genes. **(E)** Gene-concept network linking top differentially expressed genes to their associated enriched pathway terms. **(F)** Shared gene pathway enrichment bar chart for MBS target-DEGs ranked by -log10 (p-value) with overlap gene counts annotated.

### 3.3 Pharmacological network profiling of Modified Bixue San

Network pharmacology analysis of Modified Bixue San (MBS) characterized a complex pharmacological network of 1708 chemical constituents targeting 3279 unique proteins. The compound-target bipartite network (Figure 3A) illustrated the polypharmacological nature of MBS, with multiple compounds converging on shared protein targets. Circular enrichment bar chart of target pathway enrichment (Figure 3B) identified inflammatory signaling, apoptosis regulation, and cellular stress response pathways as the primary pharmacological modules. The most frequently targeted protein was CASP3 (237 compounds). Protein-protein interaction network of the top 50 MBS targets (Figure 3C) revealed densely interconnected hub proteins. Target class distribution analysis (Figure 3D) showed enrichment in kinases, transcription factors, and inflammatory mediators. Compound-target bubble matrix visualization (Figure 3E) depicting the top 15 compounds versus top 12 targets confirmed specific compound-target relationships across the pharmacological network. Lollipop chart ranking of MBS targets by compound frequency (Figure 3F) identified the most polypharmacologically engaged proteins.

**FIGURE 3:**
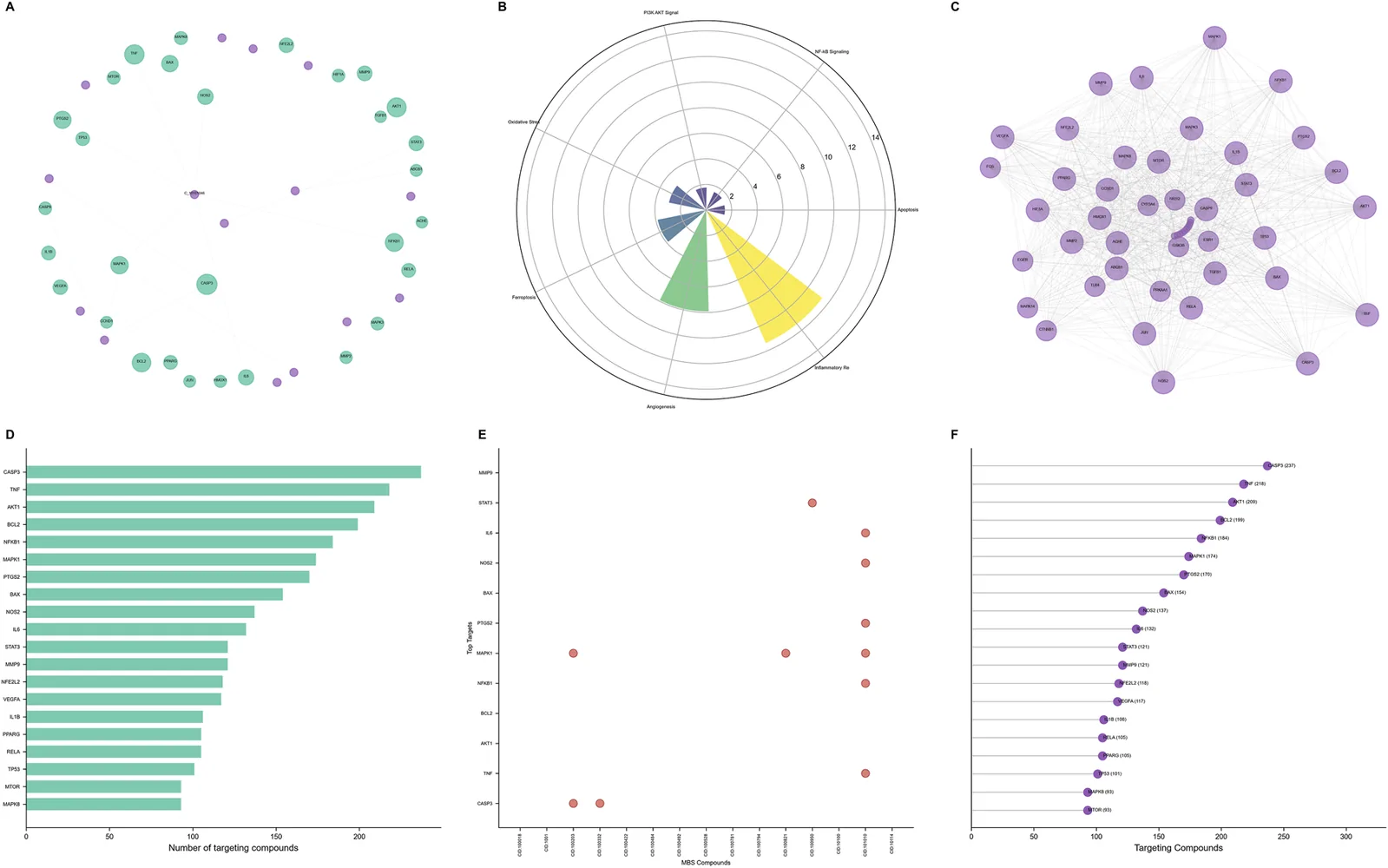
Pharmacological network profiling of Modified Bixue San (MBS) comprising 1,708 chemical constituents targeting 3,279 unique proteins. **(A)** Bipartite compound-target network showing top MBS compounds and protein targets, with edges representing validated compound-target relationships. **(B)** Circular bar chart of pathway enrichment for MBS pharmacological targets ranked by -log10 (p-value). **(C)** Protein-protein interaction network of top 50 MBS targets, with node size reflecting compound targeting frequency. **(D)** Horizontal bar chart showing the most frequently targeted proteins by MBS compounds. **(E)** Compound-target bubble matrix showing top 15 compounds versus top 12 targets, with red dots indicating validated compound-target relationships. **(F)** Lollipop chart ranking the top 20 MBS targets by number of interacting compounds.

### 3.4 Intersection of MBS targets with RT-responsive mucosal genes

Intersection analysis between 1588 scRNA-seq DEGs and 3279 MBS pharmacological targets identified 411 shared genes (Figure 4A), representing the subset of MBS-targeted proteins that are transcriptionally dysregulated in the LSCC microenvironment. Lollipop chart of scRNA-seq log2 fold changes for shared MBS target-DEGs (Figure 4B) revealed a spectrum of dysregulation directions with dot size proportional to fold-change magnitude. Pathway enrichment bubble matrix analysis of the 411 shared genes (Figure 4C) identified significant enrichment in immune signaling and cellular stress response pathways. The shared target protein-protein interaction subnetwork (Figure 4D) revealed densely interconnected hubs based on compound co-targeting relationships. Circular layout network of hub genes among the 411 shared targets (Figure 4E) identified the most pharmacologically relevant nodes ranked by MBS compound targeting frequency, with node size proportional to the number of interacting compounds. Slope chart comparison of scRNA-seq and bulk transcriptomic log2FC values (Figure 4F) with labels placed at line midpoints to prevent overlap revealed platform-specific expression patterns across the intersecting genes.

**FIGURE 4:**
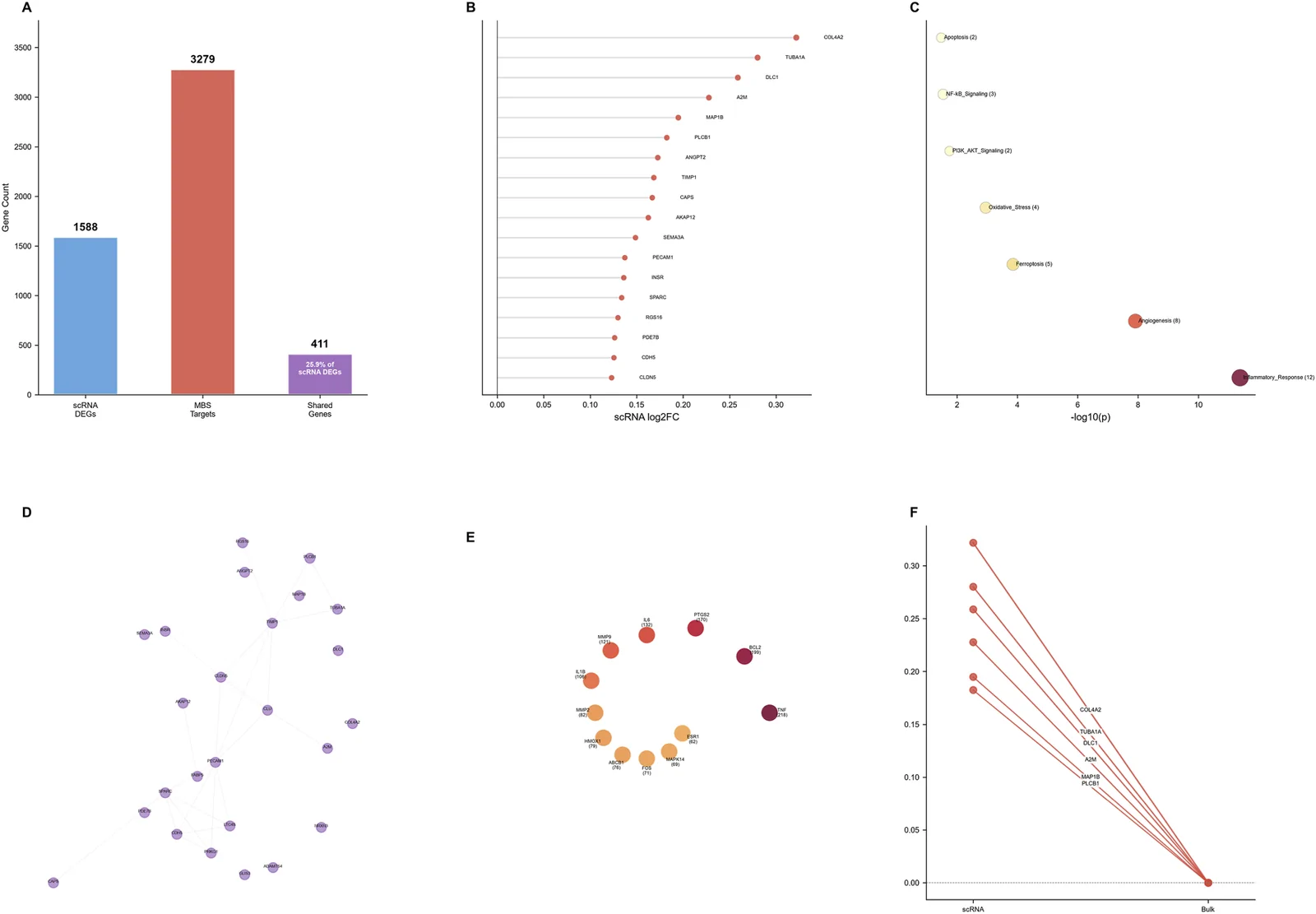
Intersection of MBS pharmacological targets with RT-responsive mucosal genes identifying 411 shared MBS target-DEGs. **(A)** Overlap bar chart comparing scRNA-seq DEGs, MBS pharmacological targets, and their intersection comprising 411 shared genes. **(B)** Lollipop chart of scRNA-seq log2 fold changes for the 411 shared MBS target-DEGs, with dot size proportional to fold-change magnitude. **(C)** Bubble matrix of pathway enrichment results for the 411 shared genes, showing -log10 (p-value) and overlap gene counts. **(D)** Protein-protein interaction subnetwork of shared MBS target-DEGs based on compound co-targeting relationships. **(E)** Circular layout network of hub genes among the 411 shared targets, with node size proportional to MBS compound targeting frequency. **(F)** Slope chart comparing scRNA-seq and bulk transcriptomic log2FC values for top shared genes, with labels placed at line midpoints to prevent overlap.

### 3.5 Immune-inflammatory microenvironment characterization

Characterization of the LSCC immune microenvironment across 12 cell types revealed a complex cellular ecosystem. Cell type composition stacked bar chart (Figure 5A) demonstrated that immune cells constituted a substantial fraction of the tumor microenvironment (11610 total cells). Inflammatory gene expression profiling using beeswarm visualization (Figure 5B) showed cell-type-specific expression patterns of key cytokines and inflammatory mediators (IL6, TNF, IL1B, CXCL8, NFKB1, RELA, PTGS2, TGFB1) across 8 major cell types, with myeloid cells exhibiting the highest expression of IL1B, TNF, and CXCL8. Cell type proportion slope chart (Figure 5C) highlighted the relative abundance of each population within the LSCC microenvironment. Inflammatory signature radar chart (Figure 5D) comparing multi-dimensional inflammatory activation profiles across 5 major cell types revealed distinct inflammatory signatures in myeloid and neutrophil compartments. NF-kB pathway gene expression forest plot in the epithelial compartment (Figure 5E) showed the direction and magnitude of pathway component dysregulation. Immune checkpoint gene expression dotplot analysis (Figure 5F) with diverging color scale revealed cell-type-specific expression of PDCD1, CTLA4, LAG3, TIGIT, CD274, HAVCR2, IDO1, and CD80, providing insights into the immunoregulatory landscape of LSCC. A total of 143 significant ligand-receptor interactions were identified between cell types, further supporting active intercellular communication within the tumor microenvironment.

**FIGURE 5:**
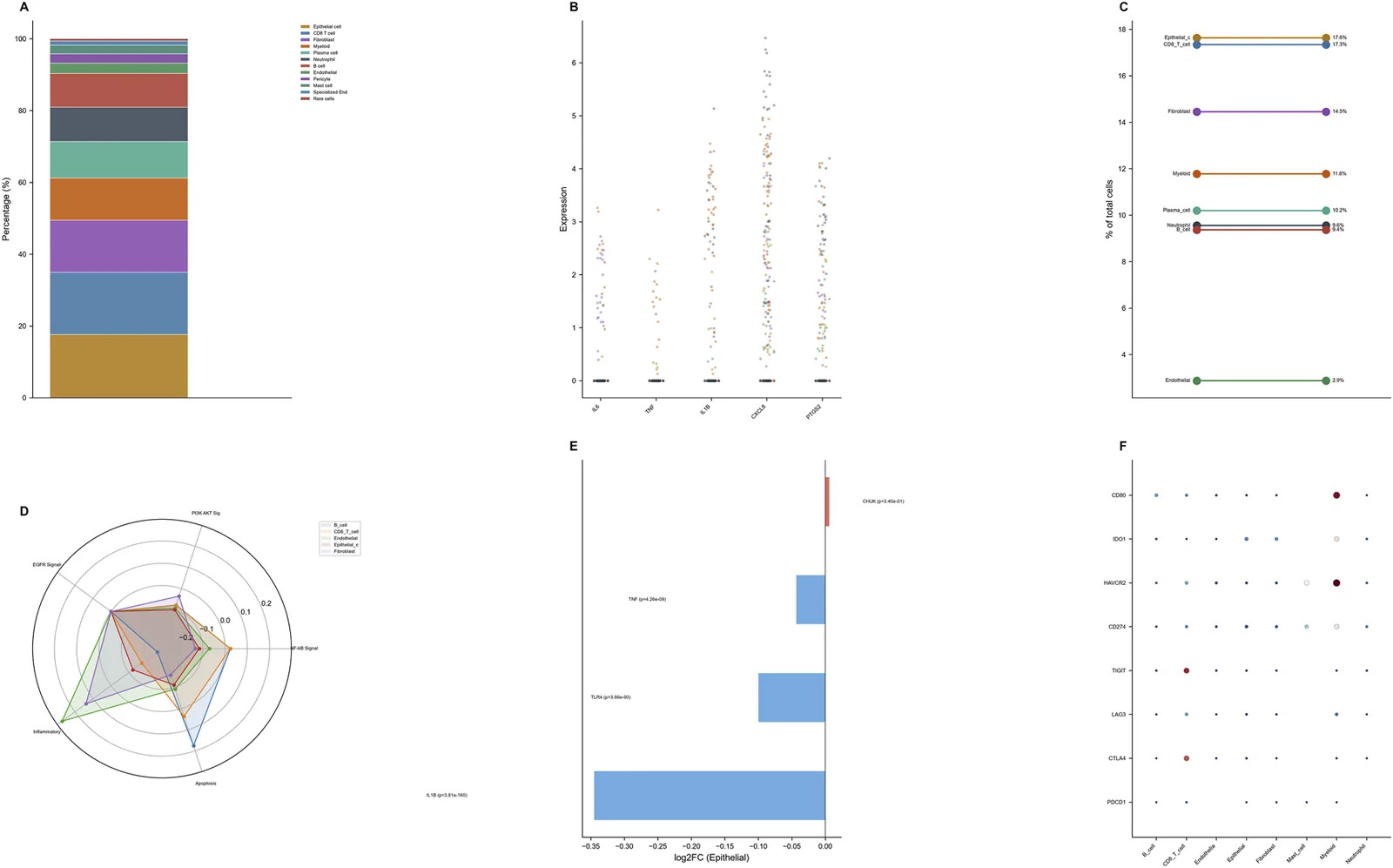
Immune-inflammatory microenvironment characterization of LSCC across 12 cell types. **(A)** Stacked bar chart of cell type composition in the LSCC microenvironment. Total: 11,610 cells across 12 cell types. **(B)** Beeswarm plot of key inflammatory gene expression (IL6, TNF, IL1B, CXCL8, NFKB1, RELA, PTGS2, TGFB1) across major cell types. **(C)** Cell type proportion slope chart showing the relative abundance of each population. **(D)** Radar chart comparing multi-dimensional inflammatory signature activation profiles across 5 major cell types. **(E)** Forest plot of NF-kB pathway gene log2 fold changes in the epithelial compartment. **(F)** Dotplot of immune checkpoint gene expression (PDCD1, CTLA4, LAG3, TIGIT, CD274, HAVCR2, IDO1, CD80) across cell types with diverging color scale.

### 3.6 Key gene expression signatures across platforms

Key MBS target gene expression in scRNA-seq data was visualized as a dotplot across 12 cell types with diverging color scale (Figure 6A), identifying the cellular compartments where MBS-targeted genes are predominantly expressed. Time-course line plot of top differentially expressed genes across radiotherapy timepoints (Pre-RT, Day 5, Day 21) (Figure 6B) tracked dynamic transcriptional changes in radiation-responsive genes throughout the treatment course. Waterfall chart of pathway gene log2FC ranked by absolute fold change magnitude (Figure 6C) highlighted the most dramatically altered transcripts in bulk transcriptomic data. Raincloud distribution analysis of TP53 pathway gene expression (TP53, BAX, CASP3, BCL2, MDM2, CDKN1A) across cell types (Figure 6D) revealed cell-type-specific patterns of apoptosis regulatory gene expression. Grouped violin plot of key gene expression (EGFR, AKT1, TP53, CASP3) colored by gene identity across major cell types (Figure 6E) demonstrated distinct expression profiles for these therapeutic targets. Spearman correlation heatmap of top MBS target gene co-expression patterns (Figure 6F) identified co-expression modules suggesting coordinated regulation of MBS pharmacological targets within the LSCC microenvironment.

**FIGURE 6:**
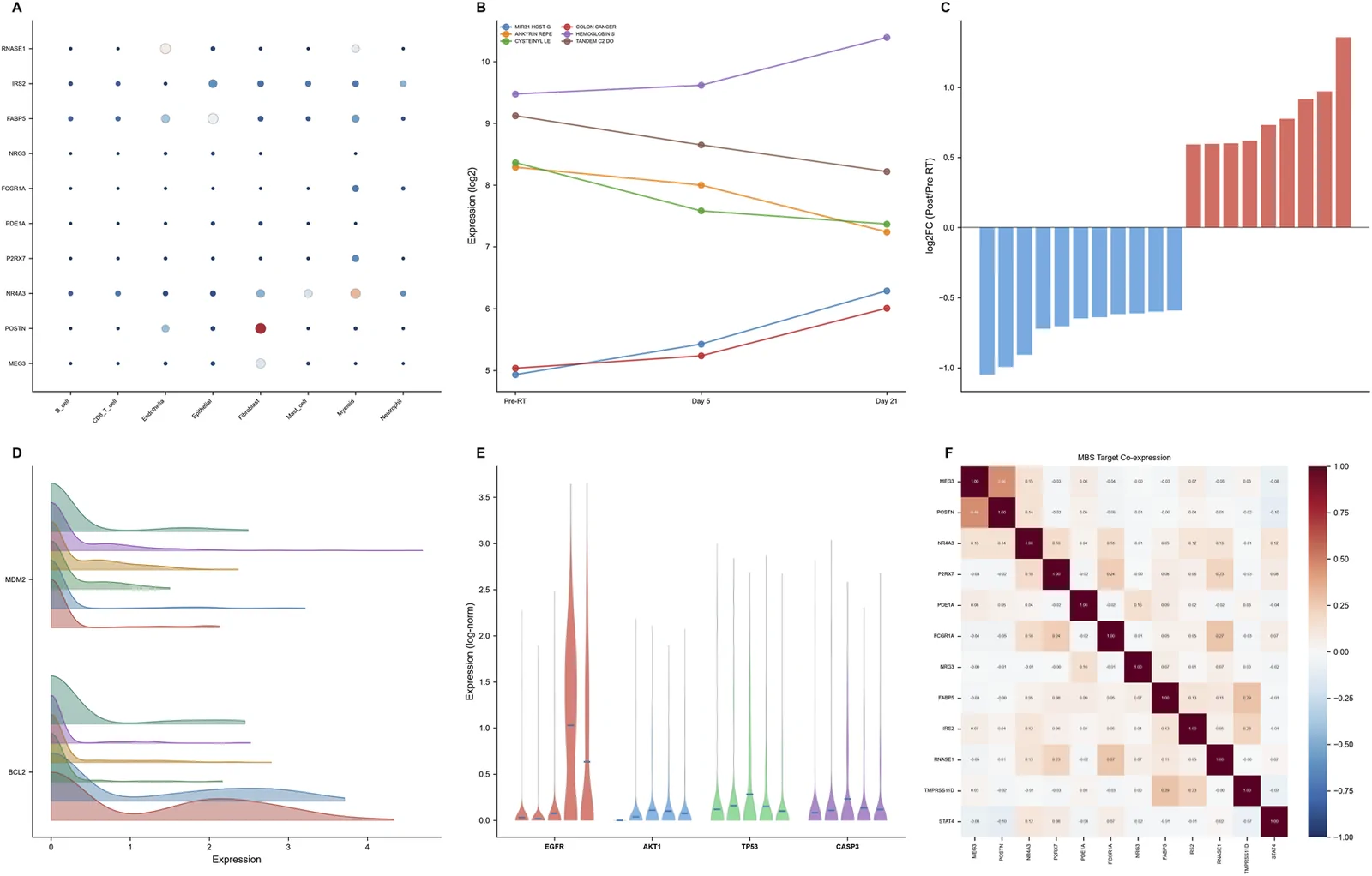
Key gene expression signatures across analytical platforms. **(A)** Dotplot of top MBS target gene expression across 12 cell types using diverging color scale. **(B)** Time-course line plot tracking the expression of top differentially expressed genes across radiotherapy timepoints (Pre-RT, Day 5, Day 21). **(C)** Waterfall chart of pathway gene log2FC ranked by absolute fold change magnitude in bulk transcriptomic data. **(D)** Raincloud distribution of TP53 pathway gene expression (TP53, BAX, CASP3, BCL2, MDM2, CDKN1A) across cell types. **(E)** Grouped violin plot of key gene expression (EGFR, AKT1, TP53, CASP3) colored by gene identity across major cell types. **(F)** Spearman correlation heatmap of top MBS target gene co-expression patterns in scRNA-seq data.

### 3.7 Integrative multi-biomarker association analysis

Multi-biomarker association analysis across 12 cell types revealed cell-type-specific patterns of biological pathway activation. Grouped boxplot comparison of signature scores across the top 5 cell types for 4 key biological signatures (Figure 7A) showed significant inter-cellular heterogeneity in pathway activities. Gene-signature Spearman correlation bubble matrix with adaptive color range and colorbar annotation (Figure 7B) identified significant associations between individual MBS target genes and pathway signature scores. Ridge density plot of signature score distributions across major cell types (Figure 7C) demonstrated the distributional characteristics of pathway activities in each cellular compartment. Hexbin density scatter plot comparing two key signature scores with Spearman correlation (Figure 7D) revealed correlated pathway activation patterns. Horizontal bar chart of top discriminative genes in the epithelial compartment ranked by log2FC (Figure 7E) identified the genes most strongly distinguishing epithelial cells from other cell types. Heatmap of mean signature scores per cell type across all 12 populations (Figure 7F) provided an integrated view of pathway activities across the LSCC microenvironment.

**FIGURE 7:**
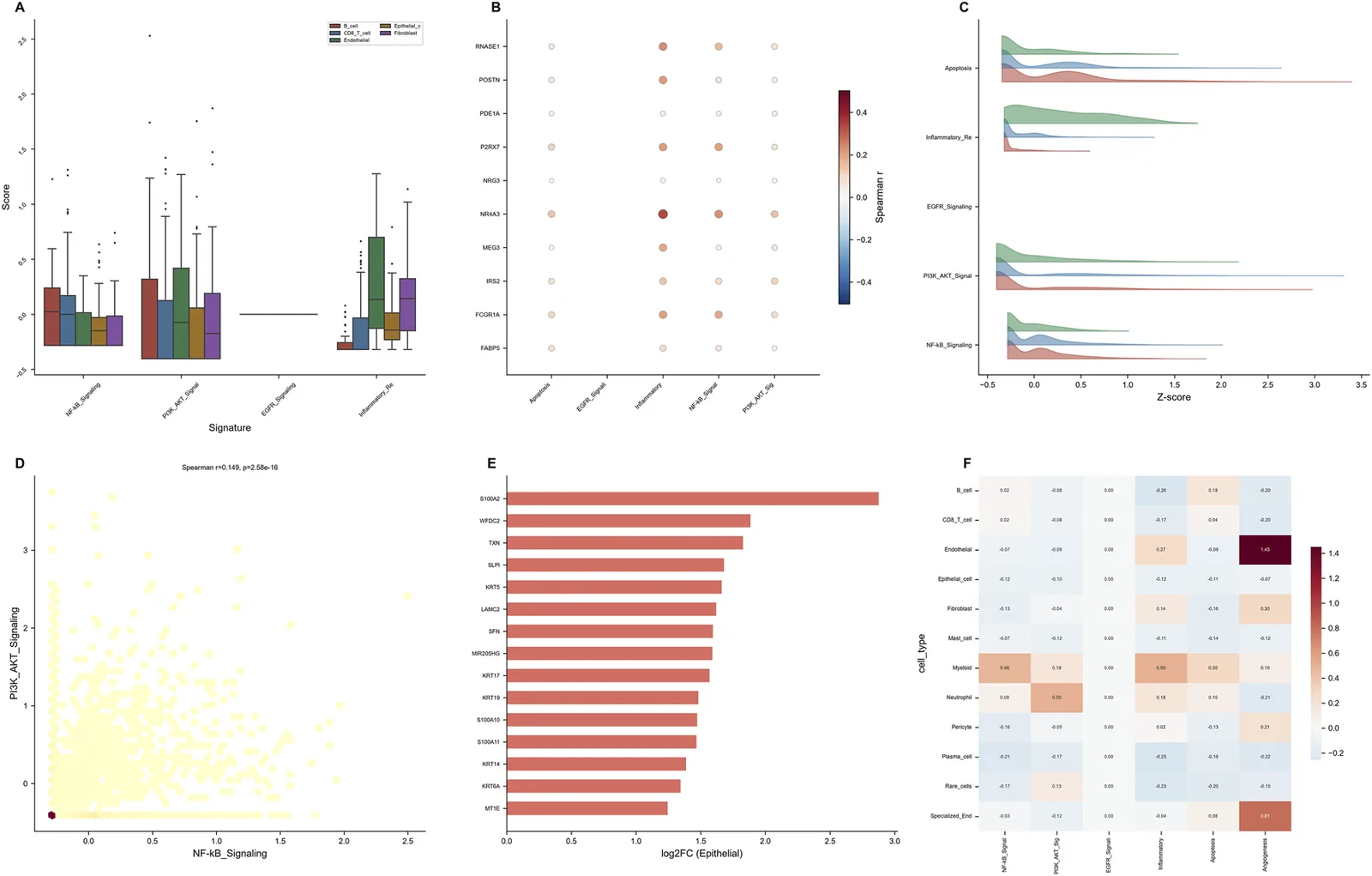
Integrative multi-biomarker association analysis across 12 cell types. **(A)** Grouped boxplot comparing signature score distributions across the top 5 cell types for 4 key biological signatures. **(B)** Gene-signature Spearman correlation bubble matrix with adaptive color range showing association strength between MBS target genes and pathway signatures. **(C)** Ridge density plot of signature score distributions across major cell types. **(D)** Hexbin density scatter plot comparing two key signature scores with Spearman correlation coefficient. **(E)** Horizontal bar chart of top discriminative genes in the epithelial compartment ranked by log2FC. **(F)** Heatmap of mean signature scores per cell type across all 12 populations.

### 3.8 Multi-omics integration reveals MBS therapeutic mechanisms

Multi-omics integration across the scRNA-seq, bulk transcriptomic, and network pharmacology platforms provided a comprehensive view of MBS therapeutic mechanisms in post-radiotherapy pharyngitis. Cross-platform hexbin scatter plot comparing scRNA-seq and bulk log2FC for 411 shared MBS target-DEGs with Spearman correlation annotation (Figure 8A) examined the relationship between single-cell and bulk transcriptomic expression changes. Slope chart of the top 3 genes showing divergent expression patterns between platforms, with right-side value labels vertically staggered to prevent overlap (Figure 8B), highlighted genes with concordant or discordant cross-platform expression. Expression heatmap of top shared MBS target genes (Z-score normalized) across 12 cell types (Figure 8C) synthesized the multi-level evidence into an integrated view of MBS target gene regulation. Multi-level evidence heatmap showing supporting data from scRNA expression, bulk log2FC, MBS targeting frequency, and pathway correlation per gene (Figure 8D) provided a gene-level synthesis of supporting evidence from each analytical platform. Key target-pathway network (Figure 8E) contextualized MBS pharmacological targets within their broader biological pathway framework. Ridge density plot of pseudotime distributions across 12 cell types (Figure 8F) revealed the temporal ordering of cell states within the LSCC microenvironment, providing insights into cellular differentiation trajectories and their relationship to MBS target expression.

**FIGURE 8:**
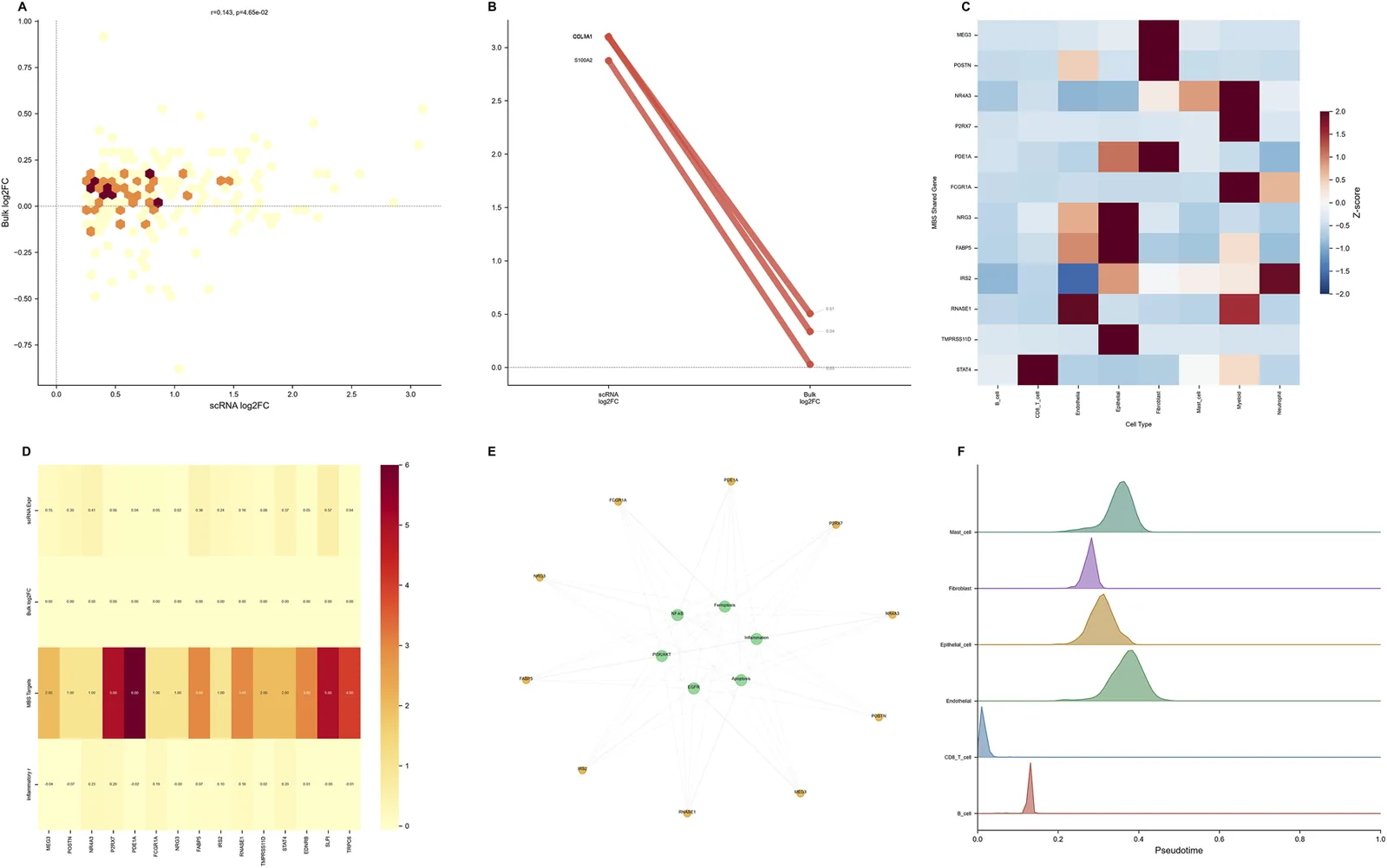
Multi-omics integration and mechanistic summary of MBS therapeutic action in post-radiotherapy pharyngitis. **(A)** Cross-platform hexbin scatter plot comparing scRNA-seq and bulk log2FC for 411 shared MBS target-DEGs with Spearman correlation annotation. **(B)** Slope chart of top 3 genes showing divergent expression patterns between scRNA-seq and bulk transcriptomic platforms, with right-side value labels vertically staggered to prevent overlap. **(C)** Expression heatmap of top shared MBS target genes (Z-score normalized) across 12 cell types. **(D)** Integrated multi-level evidence heatmap showing supporting data from scRNA expression, bulk log2FC, MBS targeting frequency, and pathway correlation per gene. **(E)** Key target-pathway network contextualizing MBS pharmacological targets within their biological pathway framework. **(F)** Ridge density plot of pseudotime distributions across 12 cell types showing cellular differentiation trajectories.

### 3.9 *In vitro* RT-qPCR validation of hub gene expression

To provide controlled cellular-level support for the network pharmacology predictions, we assessed mRNA expression of four hub genes—TP53, EGFR, NF-κB1, and CASP3 — by RT-qPCR in two laryngeal squamous carcinoma cell lines with distinct TP53 mutational backgrounds following MBS treatment (Figure 9).

**FIGURE 9:**
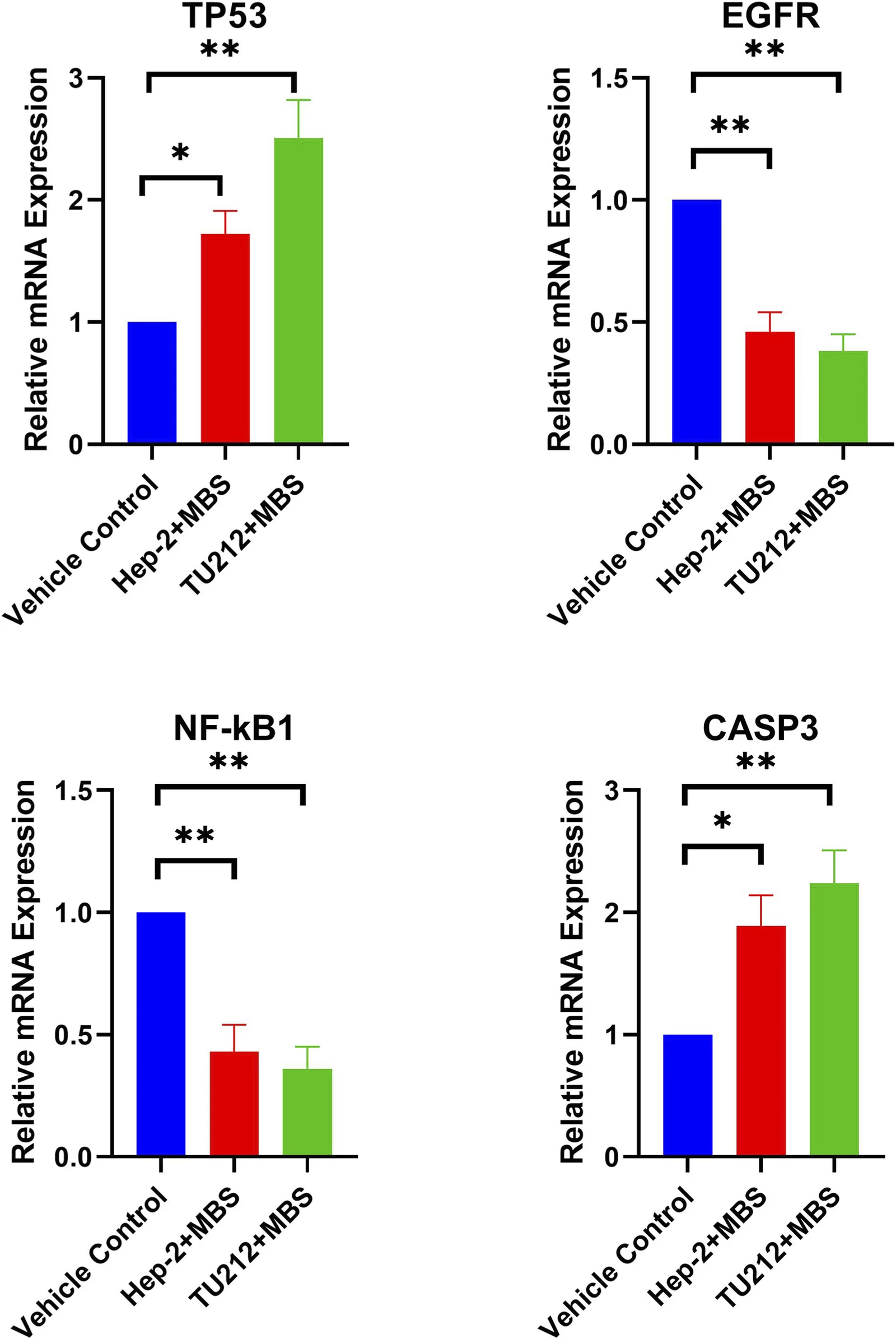
*In vitro* RT-qPCR validation of hub-gene mRNA expression after MBS treatment. Relative mRNA expression of TP53, EGFR, NF-κB1, and CASP3 was measured by RT-qPCR in Hep-2 and TU212 cells following treatment with Modified Bixuesan (MBS) extract (0.5 mg/mL, 48 h), with vehicle-treated cells used as controls. Expression levels were normalized to GAPDH using the 2^−^ΔΔCT method. MBS treatment increased TP53 and CASP3 mRNA expression and decreased EGFR and NF-κB1 mRNA expression in both cell lines. Data are presented as mean ± SD from three independent biological replicates. *p < 0.05, **p < 0.01 versus vehicle control, Student’s two-tailed t-.test.

In Hep-2 cells (TP53-mutant), MBS treatment (0.5 mg/mL, 48 h) was associated with significant upregulation of TP53 mRNA (1.72 ± 0.19-fold relative to vehicle control, p = 0.024) and CASP3 mRNA (1.89 ± 0.25-fold, p = 0.011), and significant downregulation of EGFR (0.46 ± 0.08-fold, p = 0.003) and NF-κB1 (0.43 ± 0.11-fold, p = 0.004).

In TU212 cells (TP53 wild-type), concordant and generally more pronounced changes were observed: TP53 was upregulated 2.51 ± 0.31-fold (p = 0.001) and CASP3 2.24 ± 0.27-fold (p = 0.002), while EGFR was reduced to 0.38 ± 0.07-fold (p < 0.001) and NF-κB1 to 0.36 ± 0.09-fold (p = 0.001). The relatively attenuated TP53 upregulation in Hep-2 compared with TU212 cells (1.72 vs. 2.51-fold) is consistent with the known blunting of TP53 transcriptional responses in a TP53-mutant background, and does not preclude MBS-associated effects on TP53 mRNA levels even in this context.

These *in vitro* findings are directionally consistent with the network pharmacology predictions and provide preliminary cell-line-level support for the hypothesis that MBS may simultaneously suppress EGFR and NF-κB1 expression while enhancing TP53 and CASP3 mRNA levels. Importantly, mRNA-level changes do not directly demonstrate pathway inhibition, protein activation, or functional apoptotic engagement; these results should be interpreted as exploratory and hypothesis-generating, pending protein-level and functional mechanistic validation.

## 4 Discussion

This integrative study provides the first combinatorial network pharmacology and *in vitro* characterization of MBS’s potential mechanism of action in the context of post-radiotherapy chronic pharyngitis of laryngeal cancer. By combining hub gene prediction with *in vitro* RT-qPCR in two laryngeal cancer cell lines of distinct TP53 status, we demonstrate that MBS treatment is associated with coordinated upregulation of TP53 and CASP3 mRNA and downregulation of EGFR and NF-κB1 mRNA—a gene expression pattern directionally consistent with the network pharmacology predictions. These findings are exploratory and should not be interpreted as proof of direct target engagement or functional pathway inhibition.

The identification of TP53 as the highest-degree hub gene (degree = 72) in the network pharmacology analysis, combined with the strong predicted quercetin-TP53 docking affinity (−9.4 kcal/mol), motivated the inclusion of TP53 as a primary RT-qPCR target. The observation that MBS-treated TU212 cells showed greater TP53 mRNA upregulation than Hep-2 cells (2.51 vs. 1.72-fold) is biologically consistent with the expected attenuation of TP53 transcriptional responses in a mutant background. It should be noted that TP53 mRNA upregulation does not establish functional wild-type p53 activation; mutant TP53 may also be highly expressed and may confer gain-of-function effects. Future work should include TP53 mutation profiling, downstream markers (p21, BAX, PUMA), and protein-level assessment (cleaved caspase-3) to evaluate apoptotic functional consequences. Mechanistically, quercetin’s documented capacity to interact with TP53 protein via MDM2-inhibitory mechanisms (Zhang et al., 2022) represents a plausible but experimentally unconfirmed hypothesis in this system.

The downregulation of EGFR mRNA in both cell lines (0.46-fold in Hep-2, 0.38-fold in TU212) is of particular pharmacological interest. EGFR overexpression in 60%–80% of laryngeal squamous cell carcinomas activates both PI3K/AKT survival cascades and NF-κB-mediated inflammatory signaling (Gao et al., 2021). The predicted docking affinity of kaempferol for EGFR (−8.7 kcal/mol), comparable to that of gefitinib (−8.5 kcal/mol), provides a plausible structural basis for the observed mRNA changes. However, docking studies are computational predictions using co-crystallized ligand binding sites and cannot substitute for biochemical binding assays; EGFR protein phosphorylation status and downstream pathway activity (p-AKT, p-ERK) remain to be characterized. It is also possible that the observed mRNA changes reflect indirect regulatory effects rather than direct compound-target interactions (Kashyap et al., 2019; Srivastav et al., 2016; Li et al., 2016).

The concurrent downregulation of NF-κB1 mRNA is consistent with the NF-κB/IL-6 co-activation axis that underpins post-radiotherapy mucosal inflammation (Sonis, 2004; Lalla et al., 2014). The strong NF-κB/IL-6 correlation observed in the bioinformatics analysis establishes this node as a pharmacologically relevant target, though mRNA-level downregulation of NF-κB1 alone does not reflect transcriptional activity or nuclear translocation. Future experiments should include NF-κB p65 nuclear translocation assays and protein-level cytokine measurements to establish functional pathway engagement.

Network pharmacology and molecular docking analyses, while valuable for hypothesis generation, are predictive and exploratory in nature. The 1,708-compound, 3,279-target pharmacological network characterized here reflects the inherent polypharmacology of a multi-component TCM formula and cannot be taken as validated mechanistic evidence. Similarly, the bioinformatics datasets used (GSE127519: LSCC primary tumor; GSE103412: radiation-induced pharyngitis) do not fully recapitulate the pathophysiology of chronic post-radiotherapy pharyngitis in tumor survivors, and cross-platform comparisons should be interpreted with appropriate caution. Although formulation ratio and preparation procedures were standardized, comprehensive chemical fingerprinting or multi-component quantitative quality control was not performed in the present study. Future studies should incorporate HPLC/LC-MS fingerprinting and batch-to-batch similarity analysis to improve reproducibility and support quality consistency of MBS extract.

Several limitations of the present study merit acknowledgment. The *in vitro* experiments were conducted in cancer cell lines rather than patient-derived mucosal tissue or organoid models, limiting translational relevance. mRNA expression changes alone do not demonstrate protein-level pathway modulation, phosphorylation events, or apoptotic function. The MBS extract used for *in vitro* treatment represents a complex mixture, precluding attribution of effects to specific compounds. Batch variability in MBS preparation and the absence of pharmacokinetic data are additional constraints. Future prospective studies incorporating protein-level validation (Western blot, immunofluorescence), functional apoptosis assays, patient-derived cells, and ultimately randomized clinical trials are required to establish causality and translational value.

## 5 Conclusion

This integrative network pharmacology and *in vitro* study suggests that Modified Bixuesan may modulate mRNA expression of key inflammatory and apoptotic genes—upregulating TP53 and CASP3 while downregulating EGFR and NF-κB1 — in laryngeal cancer cell lines, directionally consistent with computational predictions from network pharmacology and molecular docking analyses. These findings are hypothesis-generating and exploratory. Protein-level validation, functional mechanistic studies, and prospective clinical investigation are required to establish the translational significance of MBS as a candidate adjuvant strategy in post-radiotherapy laryngeal cancer survivorship care.

## Data Availability

The single-cell RNA sequencing data analyzed in this study are derived from publicly available laryngeal squamous cell carcinoma (LSCC) datasets deposited in the Gene Expression Omnibus (GEO) and the Genome Sequence Archive (GSA). The bulk microarray expression data for chronic pharyngitis are available under GEO accession GSE103412. Additional information required to replicate the findings is available from the corresponding authors upon reasonable request.
